# The geropathology of organ-specific aging

**DOI:** 10.15761/jts.1000458

**Published:** 2021-06-16

**Authors:** Jenna Klug, Sara Christensen, Denise M. Imai, Timothy A. Snider, Warren Ladiges

**Affiliations:** 1 Department of Comparative Medicine, School of Medicine, University of Washington, Seattle, WA, USA; 2 Department of Pathology, Microbiology and Immunology, School of Veterinary Medicine, University of California, Davis, CA, USA; 3 Department of Veterinary Pathobiology, College of Veterinary Medicine, Oklahoma State University, Stillwater, OK, USA

**Keywords:** geropathology, organ-specific aging, age-related lesions, anti-aging drug responsive organs

## Abstract

Aging is a complex multidimensional process of progressive decline affecting multiple organ systems by a number of processes that are still not well understood. While many studies have focused on the approach of studying aging across multiple organs, assessment of the contribution of individual organs to overall aging processes is under appreciated. The ability to study and compare organs in the context of organismal aging has been documented recently using a geropathology grading platform in laboratory mice. This concept consists of identifying and grading age-related histologic lesions within organs to generate a quantitative lesion score for each organ, which is representative of the presence and degree of organ-related pathology, and can be compared to scores from other organs examined. This geropathology approach provides a powerful tool to elucidate the basic mechanisms of aging in multiple organs, as well as the response of organs to therapeutic interventions. Furthermore, ongoing work with the concept has expanded and adapted the geropathology grading system to other preclinical animal model species that are commonly used to understand disease associated phenotypes in aging humans, ultimately adding to the utility of the concept.

Aging is multidimensional, involving a number of processes still not well understood. This is partly due to the complexity of specialized organs and tissues that are highly customized to perform essential functions. It is thus no surprise that aging is still a mystery. Attempts to solve this mystery are ongoing and focused mainly on organismal aging, or aging as it occurs in the whole organism. However, from the organismal perspective, the contribution of individual organs to overall aging is under appreciated. Many studies have focused on how aging and age-related diseases affect individual organs but the contribution of each organ to overall aging is largely ignored. The ability to study and compare organ aging in the context of organismal aging has recently been documented using a geropathology approach [1]. This concept consists of identifying and grading age-related histopathologic lesions so that a quantitative score is established for each organ allowing for comparison of lesion scores between all organs examined and between all animals in a specific cohort [2]. Therefore, the contribution of each organ to aging can be assessed, in contrast to studying the effect of aging or age-related disease on each organ.

The geropathology grading approach has been validated in mice as a useful tool to study aging and the relationship of age-related histopathologic lesions in specific organs to age-related disease [3]. Geropathological interrogation of individual organs provides a powerful look at the morphologic changes associated with increasing age in an organ-dependent manner. For example, based on severity of age-related histopathologic lesion scores, it can be seen that different organs age at different rates with increasing age in C57BL/6 and C57BL/6 x BALB/c (CB6F1) mice (Figure 1A). The heart ages earlier and more rapidly in CB6F1 mice from 8 months to 24 months compared to C57BL/6 mice. Surprisingly, there is no difference in aging of the lungs across this age span in the two strains. For the liver, age-related lesions are seen 8 months earlier in C57BL/6 mice and there is an increase in aging in C57BL/6 mice from 16 to 32 months. The pattern was similar for the kidney, with age-related lesions occurring earliest in C57BL/6 mice at 16 months and then progressing more rapidly.

The second example provides insight into how different organs respond to therapeutic drugs based on changes in severity of lesion scores. Studies with C57BL/6 mice treated for 3 months starting at 20 months of age have shown that organ response based on lesion scores is drug dependent in four major organs- heart, lungs, liver and kidney (Figure 1B). For rapamycin, an mTOR inhibitor, kidney, heart and liver were most responsive in males but only kidney was responsive in females using a dose of 14 ppm in the feed (4; unpublished observations). For acarbose, an antidiabetic drug, heart and kidney were most responsive in both genders at a dose of 1000 ppm in the feed (unpublished observations). For phenyl butyric acid, an inhibitor of histone deacetylation, lungs and kidney were most responsive in both genders at a dose of 1000 ppm in the feed (unpublished observations). In addition, published observations for fisetin, a natural product with senolytic activity, have shown lungs and kidney to be most responsive [5]. It is worth noting that the kidney appears to be less drug dependent, suggesting it might serve as a sentinel organ in drug studies investigating effects on aging, at least in C57BL/6 mice of both genders. These types of observations will be invaluable for helping make decisions on selection of effective drug combinations for aging intervention studies.

## Conclusion

In conclusion, geropathological assessment of individual organs from aging rodents can serve as a platform for determining contribution to organismal aging. In this respect, more insight can be obtained for assessing biological age, response to therapeutic interventions, and alignment with molecular, genetic and epigenetic features associated with increasing age. The platform has been developed for mice and recently in rats. Work is ongoing to develop a geropathology system for nonhuman primates [6]. Even though the geropathology concept is mostly applied to preclinical animal model studies, some aspects could easily be transitioned to clinical studies using human biopsy specimens or tissue banks.

## Figures and Tables

**Figure 1.**
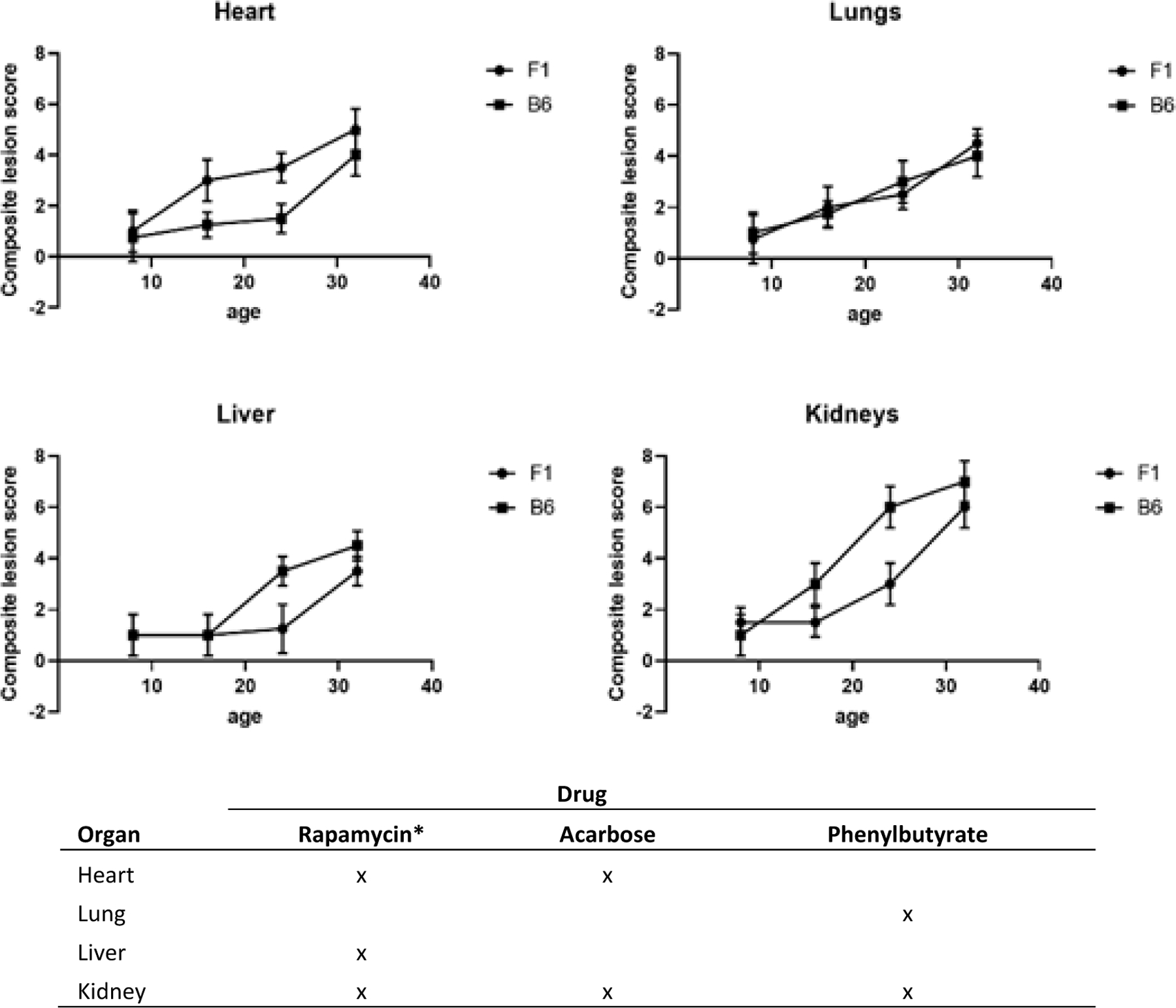
A. Composite lesion scores of heart, lungs, liver and kidney from 4 age groups of C57BL/6 and C57BL/6 x BALB/c (CB6F1) male mice show differences in the two strains at the same chronological age points. N=12/cohort. B. Drug response was determined by a significant decrease (P≤0.05) in lesion scores designated by an “X”, compared to non-medicated lesion scores over the course of 3 months in male and female C57BL/6 mice starting at 20 months of age. *For males, as females only showed drug response in the kidney. All mice were obtained from the National Institute on Aging aged rodent colony
